# Race and Pulse Oximetry in Infants With Single Ventricles Undergoing Stage 1 Palliation

**DOI:** 10.1001/jamanetworkopen.2024.5369

**Published:** 2024-04-05

**Authors:** Marcos Mills, Michelle Gleason, Michael Lin, Nikolay Braykov, Sherry Smith, Michael Fundora, Alaa Aljiffry

**Affiliations:** 1 Division of Cardiology, Department of Pediatrics, Emory University School of Medicine, Children’s Healthcare of Atlanta, Atlanta, Georgia; 2 Information Services and Technology, Children’s Healthcare of Atlanta, Atlanta, Georgia

## Abstract

This cross-sectional study investigates perioperative oxygen saturation differences in Black and White infants with single ventricles undergoing stage 1 palliation.

## Introduction

Although numerous reports have shown greater pulse oximetry inaccuracy in adults with darker skin tones, few have focused on the pediatric population.^1,2,3,4^ Even fewer reports have investigated oxygen saturation as measured by pulse oximetry (Spo_2_) in children with congenital heart disease, particularly among neonates with single ventricle anatomy undergoing stage 1 palliation (S1P).^5,6^ Among infants who underwent S1P at our center, we investigated perioperative differences between 2 commonly used bedside devices for determining arterial oxygen saturation (Sao_2_): Spo_2_ and blood gas analysis (BGA)–derived Sao_2_.

## Methods

This cross-sectional study was approved by the Children’s Healthcare of Atlanta’s institutional review board (STUDY00001501), which waived consent due to minimal risk to subjects. The STROBE reporting guideline was followed.

Patients who underwent S1P at our hospital between September 23, 2016, and January 23, 2023, were included. Race, which was self-reported by parents, was identified from the electronic health record, and patients in categories other than Black or White (9 of 132 [7%]) were excluded. Race was limited to Black and White to best compare group differences that might result from skin color. We calculated the difference between simultaneously obtained Spo_2_ measurements and calculated Sao_2_ using i-STAT blood analyzers (Abbott Laboratories) in 96 perioperative hours. Spo_2_, measured with a Masimo pulse oximeter (Masimo Corp), was obtained from stored continuous recordings and determined as the mean in the 120 seconds surrounding the automatic timestamp of BGA collection to account for transient fluctuations.

Statistical analysis was conducted using R, version 4.1.2 (R Foundation). Continuous variables are reported as medians (IQRs) and compared using Wilcoxon rank sum tests. Model development consisted of a linear mixed-effects model with repeated measurements included as a random effect and race, Spo_2_ range, and time included as fixed effects. Results are reported as estimated marginal means (EMMs). A 2-sided *P* < .05 was considered significant.

## Results

Among 123 neonates (median [IQR] age, 5 [3-7] days; 52 girls [42.3%]; 71 boys [57.7%]; 57 Black [46%]; 66 White [54%]; median [IQR] weight, 3.0 [2.7-3.4] kg) (Table), the median number of Spo_2_-Sao_2_ pairs was 22.0 (IQR, 18.0-26.5) per patient. Averaged over the levels of race and Spo_2_ range, Spo_2_-Sao_2_ discrepancy was greater preoperatively (EMM, 11.7; 95% CI, 10.9-12.6) than postoperatively (EMM, 6.3; 95% CI, 5.6-6.9). Among patients with Spo_2_ below 75% before S1P, the Spo_2_-Sao_2_ delta was greater in Black than White patients (EMM contrast, 4.7; 95% CI, 1.2-8.2; *P* = .002) (Figure). Estimated mean Spo_2_ changed from 91.3% (95% CI, 90.5%-92.1%) preoperatively to 83.3% (95% CI, 82.6%-83.9%) postoperatively (*P* < .001). The median length of stay was longer for Black patients than White patients (43.0 [IQR, 21.0-75.0] and 27.0 [IQR, 19.0-47.8] days; *P* = .06).

**Table.** Demographics and Patient Characteristics

Variable	Median (IQR)			*P* value^b^
	Total sample (N = 123)^a^	Black patients (n = 57)	White patients (n = 66)	
Sex, No. (%)				
Female	52 (42.3)	28.0 (49.1)	24.0 (36.4)	.15
Male	71 (57.7)	29.0 (50.9)	42.0 (63.6)	
Gestational age, wk	38.0 (37.0-39.0)	38.0 (37.0-39.0)	39.0 (38.0-39.0)	.56
Age at surgery, d	5.0 (3.0-7.0)	5.0 (4.0-7.0)	5.0 (3.0-6.8)	.62
Weight at surgery, kg	3.0 (2.7-3.4)	3.0 (2.6-3.3)	3.1 (2.7-3.5)	.16
CPB, min	149.5 (107.0-173.0)	153.0 (107.0-173.0)	143.0 (107.0-184.0)	.90
Unknown^c^	1	0	1	
Aortic cross clamp, min	74.0 (64.0-86.8)	76.0 (65.0-85.0)	74.0 (63.0-87.0)	.84
Unknown^c^	1	0	1	
Cerebral perfusion time, min	69.0 (61.0-76.0)	69.0 (65.0-75.0)	68.0 (57.8-79.5)	.60
Unknown^c^	42	20	22	
Shunt type, No. (%)				
Sano	71 (57.7)	33 (57.9)	38 (57.6)	>.99
BTTs	51 (41.5)	24 (42.1)	27 (40.9)	
Other^d^	1 (0.8)	0	1 (1.5)	
Length of stay, d^e^	32.0 (19.0-62.5)	43.0 (21.0-75.0)	27.0 (19.0-47.8)	.06

Abbreviations: BTT, Blalock-Taussig-Thomas shunt; CPB, cardiopulmonary bypass.

^a^ Due to limited sample size, Asian (n = 2) and multiracial (n = 6) patients were excluded from the analysis.

^b^ Pearson χ^2^ or Fisher exact and Wilcoxon rank sum tests were completed for categorical and continuous variables, respectively.

^c^ No. of patients with unknown variable.

^d^ Included hybrid and Damus-Kaye-Stansel connection with Blalock-Taussig shunt.

^e^ Defined as the time in days from stage 1 to stage 2 palliation, death, discharge home, or referral for heart transplant.

**Figure.**
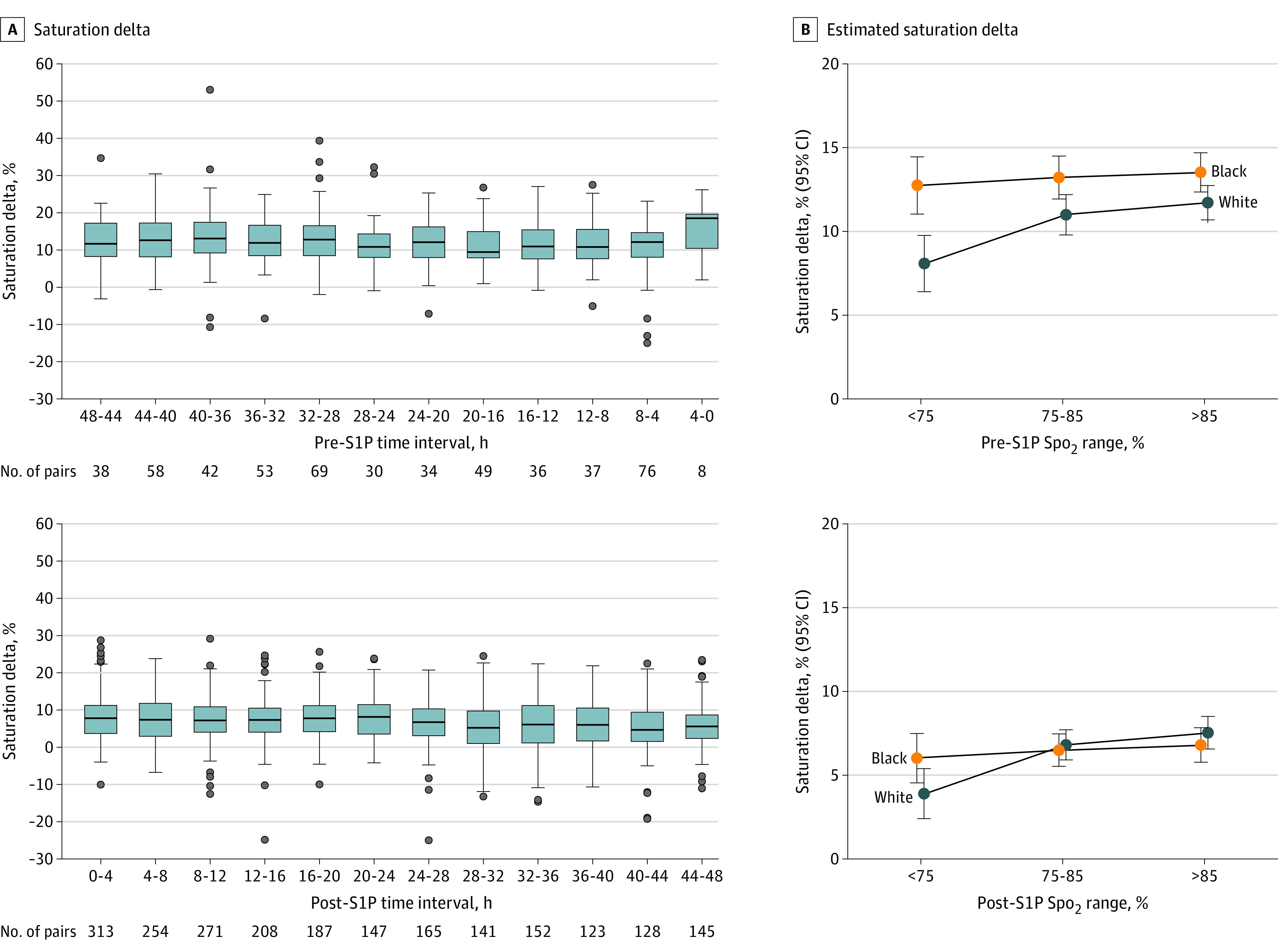
Oxygen Saturation Pairs Over Time for Black and White Patients in the 48-Hour Perioperative Period Around Stage 1 Palliation (S1P) A, Boxes indicate the total number of oxygen saturation as measured by pulse oximetry (Spo_2_)-arterial oxygen saturation (Sao_2_) pairs for each 4-hour time interval (2764 Spo_2_-Sao_2_ pairs included in the final linear mixed-effects model), with the center line indicating the median and the whiskers 1.5 times the IQR. B, Oxygen saturation pair difference adjusted for multiple measurements per patient.

## Discussion

Our findings indicate that the discrepancy between Spo_2_ and Sao_2_ was worse in the preoperative period and greater than in prior reports,^2,3,4,5^ regardless of race. We hypothesize several reasons to explain these findings: changes in the proportion of fetal hemoglobin (lowered after cardiopulmonary bypass); lower Spo_2_ levels in the postoperative period; or changes in overall cardiac output, which can affect device accuracy. We also found that although Black race was associated with significantly greater Spo_2_-Sao_2_ discrepancy at Spo_2_ less than 75% preoperatively, this was not significant at any Spo_2_ range postoperatively. Of note, Black patients had a longer length of stay, which warrants further investigation into the possible contribution of Spo_2_ inaccuracy to clinical outcomes. Finally, the discrepancy appeared consistent over time within both perioperative periods, a finding relevant to the bedside clinician and not previously reported. Although limited by the use of calculated rather than measured Sao_2_ and self-reported race, our analysis highlights the need for prospective studies investigating BGA devices in addition to Spo_2_ devices in multiple phases of care using multiple measured sample comparisons over time, a quantifiable skin tone scale, and an outcomes-based analysis.

## Notes

Supplement.
Data Sharing Statement
