# Supplementary material for: Race and Pulse Oximetry in Infants With Single Ventricles Undergoing Stage 1 Palliation
Source: JAMA Netw Open. 2024 Apr 5;7(4):e245369. doi: 10.1001/jamanetworkopen.2024.5369 (PMC10998150; doi:10.1001/jamanetworkopen.2024.5369)
Supplement: Supplement. — Data Sharing Statement [file jamanetwopen-e245369-s001.pdf]

Data Sharing Statement

Mills. Race and Pulse Oximetry in Infants With Single Ventricles Undergoing Stage 1 Palliation. *JAMA Netw Open*. Published April 05, 2024. doi:10.1001/jamanetworkopen.2024.5369

Data
Data available: Yes
Data types: Deidentified participant data
How to access data: Data available upon request to mfmill6@emory.edu
When available: With publication

Supporting Documents
Document types: Statistical/analytic code
How to access documents: Documents available upon request to mfmill6@emory.edu
When available: With publication

Additional Information
Who can access the data: Researchers and reviewers whose proposed use of the data has been approved.
Types of analyses: For specified purposes.
Mechanisms of data availability: After approval of a proposal.
